# Increased formation of neutrophil extracellular traps in patients with anti-N-methyl-d-aspartate receptor encephalitis

**DOI:** 10.3389/fimmu.2022.1046778

**Published:** 2022-12-08

**Authors:** Shan Qiao, Quan-ye Sun, Peng Zhou, Shan-chao Zhang, Zhi-hao Wang, Hai-yun Li, Ai-hua Wang, Xue-wu Liu, Tao Xin

**Affiliations:** ^1^ Department of Neurology, The First Affiliated Hospital of Shandong First Medical University & Shandong Provincial Qianfoshan Hospital, Jinan, China; ^2^ Research Center of Translational Medicine, Central Hospital Affiliated to Shandong First Medical University, Jinan, China; ^3^ Department of Neurosurgery, The First Affiliated Hospital of Shandong First Medical University & Shandong Provincial Qianfoshan Hospital, Jinan, China; ^4^ School of Medicine, Cheeloo College of Medicine, Shandong University, Jinan, China; ^5^ Department of Neurology, Qilu Hospital of Shandong University, Jinan, China; ^6^ Institute of Epilepsy, Shandong University, Jinan, China

**Keywords:** autoimmune encephalitis, anti-NMDA receptor, neutrophil extracellular traps, cytokines, neutrophils

## Abstract

**Background:**

Neutrophil extracellular traps (NETs) have been found to play an important role in several nervous system diseases. However, their role in anti-N-methyl-D-aspartate receptor (NMDAR) encephalitis remains unclear. The purpose of this study was to examine the possible role of NETs in anti-NMDAR encephalitis.

**Materials and methods:**

Eleven patients with anti-NMDAR encephalitis and ten healthy participants were enrolled. Plasma NETs levels were detected using an immunofluorescence assay and enzyme-linked immunosorbent assay. Additionally, we examined 10 plasma cytokines in patients with anti-NMDAR encephalitis and analyzed the correlation between citrullinated histone 3 levels and cytokine release.

**Results:**

Peripheral blood neutrophils from patients with anti-NMDAR encephalitis were more susceptible to NET generation. When compared with controls, cases of anti-NMDAR encephalitis showed elevated levels of IL-1 α, IL-6, IL-8, IL-13, MCP-1, and TNF-α (p < 0.05). Moreover, IL-6, IL-8, and TNF-α levels were positively correlated with H3Cit levels.

**Conclusion:**

We provide evidence that NETs may play a role in anti-NMDAR encephalitis, providing clues for elucidation of the pathogenesis of this disease.

## Introduction

Anti-N-methyl-d-aspartate receptor (NMDAR) encephalitis, which is a severe neuroautoimmune disorder, initially manifests as seizures, psychobehavioral abnormalities, and cognitive dysfunction (1). In most cases, treatment with steroids and immunoglobulins is effective (2). However, some cases are potentially fatal with patients developing intractable seizures or central hypoventilation (3). Immunologic triggers of anti-NMDAR encephalitis include tumors and viral infections (4), while approximately half of the patients with anti-NMDAR encephalitis have unknown immunologic triggers. Autopsy examination of patients with anti-NMDAR encephalitis has revealed infiltration of inflammatory cells into the perivascular, interstitial, and Virchow–Robin spaces of the brain (5). This indicates immune inflammation mediates the pathogenesis of anti-NMDAR encephalitis. However, the upstream immune and inflammatory processes that trigger anti-NMDAR encephalitis remain unclear. Accordingly, further research is warranted to elucidate the pathogenesis of anti-NMDAR encephalitis and identify novel therapeutic targets.

Neutrophils serve as the initial line of defense against pathogen invasion and play an essential role in immune response (6). When neutrophils arrive at the site of inflammation/infection, they remove pathogens by phagocytosis and the formation of neutrophil extracellular traps (NETs). NETs are large, extracellular, web-like structures, which are composed mainly of circulating free DNA (cfDNA), citrullinated histones, neutrophil proteases, and other antimicrobial proteins (7). Polymorphonuclear neutrophils (PMNs) release NETs into the extracellular space after activation and stimulation by pathogens to eradicate microbes including viruses, fungi, protozoa, and bacteria. Hyper NETosis or ineffective clearance of NETs would likely contribute to the pathogenesis of immune-related diseases (8). NETs are involved in numerous pathological processes, including infection (9), autoimmune diseases (10), tumor development (11), Alzheimer’s disease (12), acute ischemic stroke (13), peripheral nerve injury (14) and thrombosis (8). However, its role in neuroimmune diseases remains unclear. Moreover, no evidence has been found to suggest NETs are involved in anti-NMDAR encephalitis.

We aimed to investigate the potential mechanism through which NETs may trigger anti-NMDAR encephalitis and provide clues for exploring its pathogenesis and novel therapeutic targets.

## Materials and methods

This study received approval from the Ethics Committee at Qilu Hospital of Shandong University and the First Affiliated Hospital of Shandong First Medical University and was carried out. All participants and/or their legal guardian provided us with written informed consent.

### Study design and population

Eleven patients with anti-NMDAR encephalitis who were treated between June 2019 and September 2022, and ten healthy controls from the Qilu Hospital of Shandong University and the First Affiliated Hospital of Shandong First Medical University were included. Diagnosis of anti-NMDAR encephalitis was made using the diagnostic criteria given by Graus et al. (15) The inclusion criteria were as follows: (1) Rapid onset (less than 3 months) of at least one or more clinical symptoms including cognitive dysfunction, seizures, speech disturbance, movement disorder, mental disorders, decreased level of consciousness or autonomic dysfunction; (2) Anti-NMDAR-antibody-positive serum or cerebrospinal fluid (CSF); and (3) reasonable exclusion of patients with a history of other disorders. Patients with missing data were excluded. All serum samples were collected from patients during the active disease state prior to immunotherapy. As controls, healthy blood donors were recruited from health check-up clinics.

A full medical history, results of serum and CSF analyses, brain magnetic resonance imaging scans, electroencephalograms, and details regarding treatments and prognoses were documented. Autoantibodies to NMDAR, contactin-associated protein-like 2, LGI1, AMPA1, GABA-B receptor, and AMPA2 in both the serum and CSF were evaluated using an indirect immunofluorescence protocol following the manufacturer’s instructions (Euroimmun, Germany).

### Isolation of neutrophils

Human neutrophils were extracted using a neutrophil separation solution kit (LZS11131; TBDsciences, China) and density gradient centrifugation. In all studies, neutrophil purity and viability were greater than 98%. Briefly, EDTA-anticoagulated whole-blood samples were carefully layered on top of the separation solution. The neutrophil layer in the separation fluid was gently aspirated using a pipette after centrifugation for 25 minutes at 550 g and 25 °C. Red blood cell lysis buffer was added in case there were mixed red blood cells. The cells were washed with washing buffer and resuspended in RPMI-1640 medium.

### Stimulation of NETs in human neutrophils *in vitro*

Human neutrophils isolated from control individuals were treated with 10% plasma isolated from anti-NMDAR encephalitis patients or control individuals for 3-4 hours in a 5% CO2 incubator at 37°C.

### Quantification of NETs by immunofluorescence

Cells were prepared as previously reported (16, 17). Isolated neutrophils were resuspended in RPMI-1640 media at a concentration of 1 × 10^6^ cells/mL. After that, 50 µL of suspension was pipetted into each well of a 24-well plate with a poly-L-lysine-coated 12-mm glass coverslip. The plate was then incubated for 3 hours in a CO_2_ incubator at 37°C. After 30 minutes of fixation in 4% paraformaldehyde, materials were permeabilized for 1 minute with Triton X-100 (0.5%) and blocked for 1 hour with 5% bovine serum albumin. NETs were stained with anti-Neutrophil Elastase (ab254178, Abcam, UK) and anti-citrullinated histone 3 (H3Cit) (ab5103, Abcam, UK). Subsequently, Alexa Fluor 488- and Alexa Fluor 555-conjugated secondary antibodies (A0423/A0460, Beyotime Biotechnology, China) were used to incubate the samples. DNA was labeled with DAPI (C1005, Beyotime Biotechnology, China). Coverslips were mounted using a mounting solution on glass slides and photographed using a Panoramic MIDI digital slide scanner (3DHISTECH, Hungary). A random sample of five fields was used in each experiment to count neutrophils releasing NETs, which was expressed as a percentage of total neutrophils.

### Measurement of cell-free DNA

Plasma was isolated from EDTA-anticoagulated whole blood samples after centrifugation at 3000 rpm for 10 min. cfDNA was detected using a Quant-iT™ PicoGreen™ Assay Kit (P7589, Invitrogen, USA), according to the manufacturer’s instructions. Briefly, 100 μL of plasma and 100 μL of Quant-iT™ PicoGreen™ dsDNA Reagent were added to the microplate wells. The plate was incubated at room temperature for 2-5 minutes in the dark. A SpectraMax i3x microplate reader (Molecular Devices, CA, USA) was used to detect sample fluorescence at the standard fluorescein wavelength (excitation, 480 nm; emission, 520 nm). Finally, the standard curve was used to determine the DNA concentration of the samples.

### Measurement of citrullinated histone 3

H3Cit levels were determined using the Human Acetylated histone H3 (AH3) ELISA Kit (JL10987, Shanghai Jianglai Industrial Limited by Share Ltd, China) following the manufacturer’s instructions. In brief, plasma was added to the wells of a 96-well microtiter plate coated with anti-H3Cit antibody at a 1:1 dilution and incubated at 37°C for 1 hour. The liquid was not washed before adding a biotinylated antibody working solution for 1 hour at 37°C. The enzyme conjugate working solution was added after washing and incubated for 30 minutes at 37°C. Finally, we administered TMB to each well for 15 minutes at 37°C before taking instantaneous measurements with a SpectraMax i3x microplate reader (Molecular Devices, CA) at 450 nm.

### Measurement of serum cytokine levels

The serum levels of various cytokines were evaluated using commercial assays (Human Inflammation Array 1, RayBiotech, USA) following the manufacturer’s instructions. We analyzed the following cytokines: IFN-γ, IL-1 α, IL-1β, IL-10, IL-13, IL-4, IL-6, IL-8, monocyte chemotactic protein (MCP)-1, and tumor necrosis factor alpha (TNF-α). An InnoScan 300 microarray scanner (Innopsys, France) was used to visualize signals. The RayBio Q-Analyzer Tool (QAH-INF-3-SW) was used to convert the data to concentrations.

### Statistical analyses

GraphPad Prism 8.0 or SPSS v.26.0 software was used for statistical analysis. Variables with non-normal distributions are reported as the median and interquartile ranges (IQRs). Unpaired t-tests were utilized where needed. Between-variable correlations were analyzed using Spearman’s correlation coefficients. Statistical significance was set at p < 0.05 (two-sided).

## Results

### Participants

In terms of age or gender, there were no significant differences between groups. **Table 1** lists the major clinical and laboratory parameters of both groups as well as entailing information regarding the participants age and sex.

**Table 1:** Participant characteristics by groups.

Characteristics	Control group (n=10)	Anti-NMDAR encephalitis group (n=11)	*p* value
Sex (male/female)	5/5	5/6	0.590
Age, y, median (IQR)	25.25 (31.5-36.5)	21 (27-35)	0.396
mRS score at onset, median (range)	–	5 (4–5)	
Clinical syndrome, n (%)			
Prodromal infection symptoms	–	6 (54.55)	
Seizures	–	9 (81.82)	
Psychiatric abnormalities	–	7 (63.64)	
Cognitive impairment	–	7 (63.64)	
Speech disorders	–	2 (18.18)	
Decreased level of consciousness	–	2 (18.18)	
Movement disorder	–	0	
Complicated with tumors	–	0	
Ancillary tests results			
Peripheral blood WBC (× 10^9^/L), median (IQR)	6.138 (7.595-8.268)	7.55 (9.85-14.09)	0.011*
Peripheral blood neutrophil (× 10^9^/L), median (IQR)	3.733 (4.555-5.203)	5.49 (6.84-11.99)	0.004*
Peripheral blood Lymphocyte (× 10^9^/L), median (IQR)	1.695 (2.05-2.718)	1.59 (1.81-2.03)	0.146
NLR	1.318 (1.97-2.57)	2.72 (4.09-7.17)	0.009*
Treatment and prognosis, n (%)			
Steroid	–	11 (100)	
Intravenous immunoglobulin	–	8 (72.73)	
Steroid + intravenous immunoglobulin	–	10 (75)	

NMDAR, N-methyl-D-aspartate receptor; IQR, interquartile range; mRS, modified Rankin Scale; WBC, white blood cells; NLR, neutrophil-lymphocyte ratio; MRI; magnetic resonance imaging; EEG, electroencephalogram, *p value in unpaired t-test.

### Peripheral blood neutrophils from patients with anti-NMDAR encephalitis are more prone to NET formation

**Figure 1:**
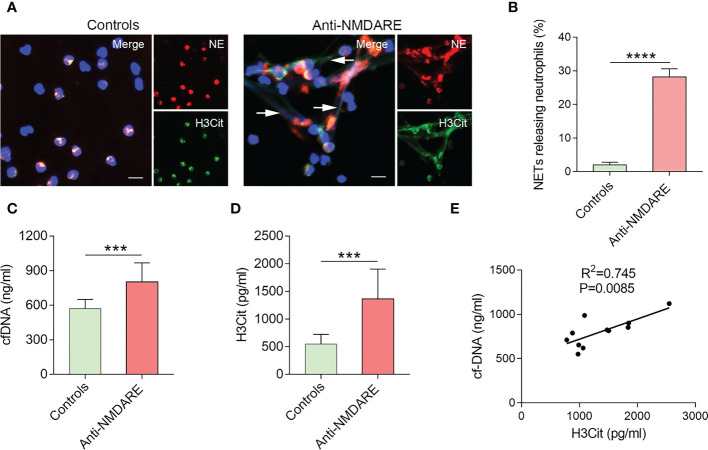
NETs accumulation in patients with anti-NMDAR encephalitis. **(A)** Representative immunofluorescence images of neutrophils releasing NETs from patients with anti-NMDAR encephalitis and healthy controls. NETs are distinguished by NE (red), H3Cit (green), and DNA (blue). NETs were denoted with arrows. Five distinct experiments are shown by representative photos. **(B)** Percentage of NET-releasing neutrophils among neutrophils isolated from the different groups. **(C)** Plasma cfDNA levels were evaluated in healthy controls (n = 10) and patients with anti-NMDAR encephalitis (n = 11). **(D)** Plasma H3Cit levels were determined in healthy controls (n = 10) and patients with anti-NMDAR encephalitis (n = 11). **(E)** Correlation between cfDNA and H3Cit levels in patients with anti-NMDAR encephalitis. Scale bar: 50 mm. ***p < 0.001, ****p < 0.0001.

To assess the ability of neutrophils to generate NETs from anti-NMDAR encephalitis patients or healthy controls, neutrophil elastase (NE), H3Cit and cfDNA NETs-specific markers were used. Immunofluorescence microscopy showed a significant difference in the number of NET-releasing cells from anti-NMDAR encephalitis patients compared with the control group (**Figure 1A**), and peripheral blood neutrophils from anti-NMDAR encephalitis patients are more prone to generate NETs (p < 0.0001) (**Figure 1B**). Additionally, patients with anti-NMDAR encephalitis had greater plasma levels of cfDNA (p = 0.0008) and H3Cit (p = 0.0002) as compared to controls (**Figure 1C, D**). In addition, cfDNA levels showed a significant positive correlation with that of H3Cit in anti-NMDAR encephalitis patients (R^2^=0.745, P = 0.0085) (**Figure 1E**).

### The inflammatory environment in anti-NMDAR encephalitis promotes the release of NETs

**Figure 2:**
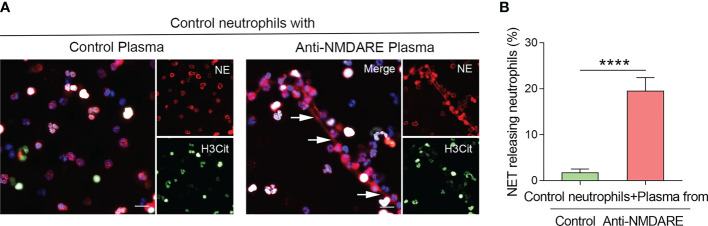
Plasma derived from anti-NMDAR encephalitis patients activates NETs generation in neutrophils. **(A)** Representative immunofluorescence photos of NET formation in healthy neutrophils treated with plasma from control and anti-NMDAR encephalitis samples. NE (red), H3Cit (green), and DNA (blue) were used to characterize NETs. NETs were indicated using arrows. Representative photos from the five distinct experiments are given. **(B)** The proportion of NET-releasing neutrophils among control neutrophils incubated with plasma from each group. Scale bar: 50 mm. ****p < 0.0001

To determine whether the inflammatory milieu of anti-NMDAR encephalitis causes NET release, we used plasma derived from anti-NMDAR encephalitis patients to stimulate healthy neutrophils. Compared to control plasma, healthy neutrophils exposed to anti-NMDAR plasma exhibited significant NETs production (**Figure 2A**), and the percentage of NET-releasing PMNs was higher (*p* < 0.0001) (**Figure 2B**).

### Correlation between H3Cit and cytokine levels in patients with anti-NMDAR encephalitis

**Figure 3:**
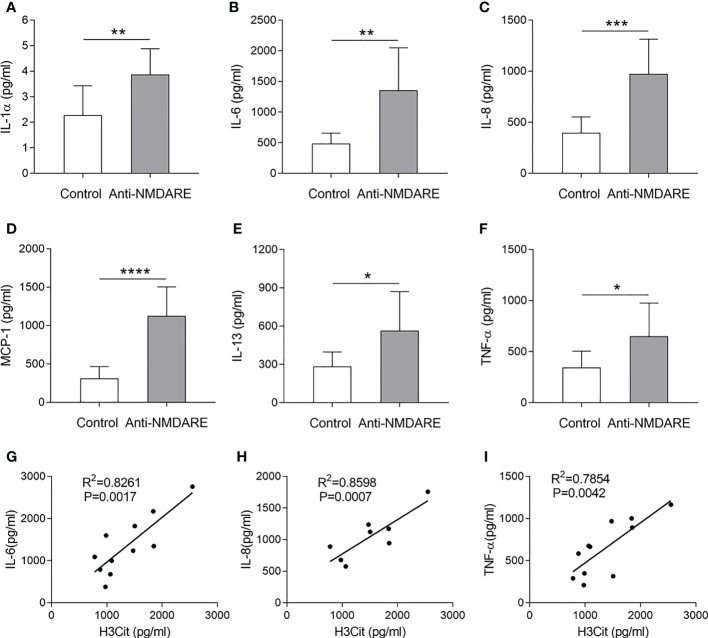
Correlation between H3Cit and cytokine levels in patients with anti-NMDAR encephalitis. Serum IL-1 α **(A)**, IL-6 **(B)**, IL-8 **(C)**, MCP-1 **(D)**, IL-13 **(E)**, and TNF-α **(F)** levels were detected in the different groups. Correlation of plasma H3Cit levels with plasma IL-6 **(G)**, IL-8 **(H)**, and TNF-α **(I)** levels in patients with anti-NMDAR encephalitis (n = 11). *p < 0.05, **p < 0.01, ***p < 0.001, ****p < 0.0001.

Previous studies have suggested that several cytokines mediate NET production. To further explore the potential underlying mechanism of NETs in anti-NMDAR encephalitis, we examined plasma levels of the aforementioned cytokines in samples from patients with anti-NMDAR encephalitis to determine their correlation with H3Cit levels. Compared to controls, patients with anti-NMDAR encephalitis had higher levels of IL-1α, IL-6, IL-8, IL-13, MCP-1, and TNF-α levels (p < 0.05). Moreover, the levels of IL-6 (R^2^ = 0.8261, P = 0.0017), IL-8 (R^2^ = 0.8598, P = 0.0007), and TNF-α (R^2^ = 0.7854, P = 0.0042) positively correlated with H3Cit levels (**Figure 3**).

## Discussion

As innate immune phagocytes, neutrophils play an important role in the immune system against pathogens. NETs are implicated in autoimmune illnesses and other non-infectious pathological processes, including as thrombosis, atherosclerosis, coagulation disorders, and cancer, in addition to their crucial function in the neutrophil innate immune response (6, 18–20). NETs’ possible significance in anti-NMDAR encephalitis has hitherto gone unexplored. As far as we know, the present study is the first to show NET development in patients with anti-NMDAR encephalitis.

Recently, NETs have been found to be released in a variety of neurological illnesses, including Alzheimer’s disease (AD), venous thrombosis, CNS infections, and stroke (8, 12, 13, 21–23). An increasing body of evidence suggests that excessive NETs release can stimulate immune cell activation and trigger neuronal damage. CSF samples from children and adults with CNS infections contained elevated neutrophil activation and active NET formation, according to Daniel et al. (21). Additionally, Vaibhav et al. reported that NET formation exacerbated acute neurological injury after traumatic brain injury (TBI) and that Toll-like receptor 4 mediated post-TBI NET formation and cerebrovascular dysfunction (17). Furthermore, Pietronigro et al. also identified intravascular neutrophil adherence and NET release in AD patients, implying that intravascular NETs may be associated with CNS damage (12).. These findings indicate that NETs may play a role in neuroimmune regulation. Despite past studies indicates that B cells play a significant part in the disease’s etiology, the mechanism of anti-NMDAR encephalitis has not been fully understood (24, 25). Autopsy and pathological examination of patients with anti-NMDAR encephalitis has revealed infiltration of numerous inflammatory cells into the brain parenchyma and more than a third of anti-NMDAR encephalitis patients have a pre-infection history (26). Evidence for the role of NETs in inflammation/infection and autoimmune disease suggests that NETs exert immunomodulatory effects through activation and differentiation of macrophages, T cells, B-cell, and dendritic cells (27, 28). Accordingly, we hypothesize that neutrophils are implicated in the pathogenesis of anti-NMDAR encephalitis. In our investigation, peripheral blood neutrophils from individuals with anti-NMDAR encephalitis were more likely to generate NETs, suggesting that neutrophil activation and NETs are important in the process of anti-NMDAR encephalitis. This provides new insights into the disease pathophysiology and prospective treatment targets.

Cytokines act as intercellular messengers in inflammatory processes. Furthermore, different cytokine subsets play regulatory roles in different types of inflammatory responses (29, 30). There is increasing evidence that cytokines/chemokines are key regulatory factors in the pathogenesis of anti-NMDAR encephalitis and are potential biomarkers for disease diagnosis and progression (31, 32). Cytokine profiles differ between patients with anti-NMDAR encephalitis and healthy controls without autoimmune neurological disease. In a study on 167 individuals, Leypoldt et al. observed that 70% of patients with early-stage anti-NMDAR encephalitis had greater CXCL13 levels than healthy controls, implying that CXCL13 could be a potential biomarker of treatment responsiveness in these patients (33). Several other cytokines are involved in T cell recruitment and proliferation, including IL-7, TNF-α, IL-10, IL-17α, IFN-γ, and CXCL10 (30–32, 34), whose levels are increased in the serum or CSF during the acute phase of anti-NMDAR encephalitis. NETs may promote the production of cytokines in various immune and inflammatory processes and thus mediate the occurrence and development of inflammation (6, 9, 12), whereas cytokines may trigger NET release to mediate disease progression. Patients with AD showed considerably greater amounts of IL-8, TNF-α,thrombin, and IL-1β in their brain microvasculature than controls, suggesting these endothelial molecules may stimulate NETs formation by adhering to neutrophils (12). Patients with moderate cognitive impairment had greater blood IL-1 levels than controls, indicating that IL-1 may induce the production of NETs and contribute to the beginning of AD (35). These findings imply that the interplay of inflammatory markers and NETs are widely involved in the incidence and progression of neurological and immune system illnesses.

In our study, patients with anti-NMDAR encephalitis had higher levels of IL-1α, IL-6, IL-8, IL-13, MCP-1, and TNF-α than controls. Furthermore, IL-6, IL-8, and TNF- levels were found to be favorably associated to H3Cit levels. TNF-α, a key mediator in immune and inflammatory responses, stimulates neutrophils and lymphocytes, increases vascular endothelial cell permeability, and triggers the production and release of other cytokines (36). IL-6 can induce B-cell differentiation to promote the inflammatory response. IL-8 stimulates chemotaxis of neutrophils and T lymphocytes, promoting the degranulation of neutrophils, causing damage to endothelial cells. Vasculature in the brain of patients with anti-NMDAR encephalitis have extensive infiltration of inflammatory cells as well as increased levels of TNF-α, IL-6, IL-8 and other inflammatory factors compared to healthy controls (29, 37, 38). This implies that inflammatory factors play a role in the development of anti-NMDAR encephalitis. Furthermore, all three inflammatory factors were found to be positively linked with NETs production, suggesting that inflammatory factors may be implicated in NETs formation during the process of anti-NMDAR encephalitis. However, additional research is needed to understand the underlying mechanisms of action.

A limitation of this study is the small sample size. Therefore, a larger sample size is required to better evaluate the diagnostic and prognostic utility of NETs in patients with anti-NMDAR encephalitis. In addition, given the risk of performing lumbar puncture, we could not obtain CSF samples which limited our ability to examine NETs release in CSF. Future large-scale, multicenter, and prospective studies are warranted to explore and clarify the underlying mechanisms and targets.

## Conclusion

Our findings indicate that peripheral blood neutrophils from patients with anti-NMDAR encephalitis are more likely to produce NETs. Furthermore, our data suggests that NETs may play a role in the process of the disease, which sheds light on the role of neutrophils in the pathogenesis of anti-NMDAR encephalitis. However, more research is needed to determine the precise underlying mechanism of action.

## Data availability statement

The original contributions presented in the study are included in the article/supplementary material. Further inquiries can be directed to the corresponding authors.

## Ethics statement

An Ethics Committee at Shandong University approved the study (No. KYLL-202008-044), which was carried out in accordance with the Declaration of Helsinki. The patients/participants provided their written informed consent to participate in this study.

## Author contributions

TX and X-WL conceived the study and supervised this work. SQ conceived the study, organized and statistical data, and drafted the manuscript. Q-YS and PZ performed the research and organized datas. S-CZ, Z-HW, H-YL, and A-HW assisted in analyzing the data. All authors contributed to the article and approved the submitted version.
